# Apolipoprotein-mediated regulation of lipid metabolism induces distinctive effects in different types of breast cancer cells

**DOI:** 10.1186/s13058-020-01276-9

**Published:** 2020-04-22

**Authors:** Céline Ben Hassen, Jorge L. Gutierrez-Pajares, Cyrille Guimaraes, Roseline Guibon, Michelle Pinault, Gaëlle Fromont, Philippe G. Frank

**Affiliations:** 1 INSERM N2C UMR1069, University of Tours, 37032 Tours, France; 2 Department of Pathology, CHRU Tours-University of Tours, Tours, 37032 France

**Keywords:** Lipids, Cholesterol, Cancer progression

## Abstract

**Background:**

The highest incidence of breast cancer is in the Western world. Several aspects of the Western lifestyle are known risk factors for breast cancer. In particular, previous studies have shown that cholesterol levels can play an important role in the regulation of tumor progression.

**Methods:**

In the present study, we modulated cholesterol metabolism in the human breast cancer cell lines MCF-7 and MDA-MB-231 using a genetic approach. Apolipoprotein A-I (apoA-I) and apolipoprotein E (apoE) were expressed in these cell lines to modulate cholesterol metabolism. The effects of these apolipoproteins on cancer cell properties were examined.

**Results:**

Our results show that both apolipoproteins can regulate cholesterol metabolism and can control the epithelial-to-mesenchymal transition process. However, these effects were different depending on the cell type. We show that expressing apoA-I or apoE stimulates proliferation, migration, and tumor growth of MCF-7 cells. However, apoA-I or apoE reduces proliferation and migration of MDA-MB-231 cells.

**Conclusions:**

These data suggest that modulating sterol metabolism may be most effective at limiting tumor progression in models of triple-negative cancers.

## Introduction

The highest incidence of breast cancer is in the Western world. Several aspects of Western lifestyles are known risk factors for breast cancer. Diets high in saturated fat, early menarche, low parity, and advanced age at first pregnancy are established risk factors for breast cancer [1]. All of these risks are more common in Western countries. Importantly, only 10% of breast cancer cases have a genetic origin [1]. As a consequence, in the Western world, environmental factors appear to have a far greater contribution to breast cancer incidence than genetic factors. Many studies have now suggested an important role for several dietary nutrients in the progression and development of breast cancer [2]. Epidemiological studies have suggested that elevated plasma cholesterol levels are associated with an increased risk of mortality and increased risk of breast cancer after menopause. In pre-menopausal women, recent reports have proposed a protective role for high-density lipoprotein (HDL) against breast cancer [3, 4]. This observation is consistent with studies showing that the use of lipid-lowering drugs in older women is associated with a reduction in breast cancer development [5]. Therefore, modulating cholesterol metabolism in breast cancer cells may alter cancer progression.

Our previous studies have shown that increased plasma cholesterol levels are associated with increased tumor burden and accelerated tumor formation in a mouse model of breast cancer (PyMTTg). They also indicate that the metabolism of cholesterol and plasma lipoproteins is abnormal in PyMTTg mice that spontaneously develop tumors of the mammary gland [6]. However, the relationship between cholesterol/lipoproteins and the formation of tumors is still poorly understood. It is therefore essential to understand how the metabolism of plasma cholesterol and, more particularly, that of the lipoproteins that carry it can affect breast cancer. While cholesterol entry may promote tumor formation in human breast cancer cells, tumor cholesterol reduction may prevent the development of tumors or slow down their development. Supporting this hypothesis, we have studied the role of the membrane receptor SR-BI, which is a transmembrane protein regulating cholesterol metabolism [7]. SR-BI binds to HDL and is responsible for the selective uptake of HDL-cholesteryl esters into the cell. Previous studies have shown that SR-BI expression is increased in tumor tissues obtained from patients with breast cancer. On the other hand, the expression of SR-BI is weak in the corresponding non-cancerous tissues [8]. We have shown that elimination of SR-BI via an shRNA approach or its pharmacological inhibition allows to reduce proliferation, rate of invasion, and development of xenograft tumors from the human breast cancer lines MCF-7 and MDA-MB-231 in immunodeficient mice [9]. This effect appears to be independent of the presence of the estrogen receptor since the cells used were either triple-negative (MDA-MB-231) or ER+ (MCF-7).

Apolipoprotein A-I (apoA-I) and apolipoprotein E (apoE) are key proteins that have been shown to regulate cholesterol metabolism in various cells. For example, both proteins have been shown to promote cholesterol efflux in macrophages, which play an important role in the development of atherosclerosis. As a consequence, macrophage expression of apoA-I or apoE have been shown to lead to reduced atherosclerosis in animal models [10, 11]. To better understand the mechanism by which cholesterol regulates tumor progression, we expressed apoA-I and apoE in the human breast cancer cell lines MCF-7 and MDA-MB-231.

## Material and methods

### Materials

The following antibodies were used: apoA-I (5F6) and apoE (1D7) antibodies were a kind gift from Dr. Yves L. Marcel (University of Ottawa, ON, Canada). Caveolin-1 was from Santa Cruz Biotechnology, Inc. (Heidelberg, Germany). Anti-mouse secondary antibody was from Fisher Scientific (Illkirch-Graffenstaden, France), and anti-rabbit secondary antibody was from BD Biosciences (San Jose, CA). All other reagents were of analytical grade.

### Cell culture

MCF-7 and MDA-MB-231 cells were obtained from the American Tissue Culture Collection (ATCC) (via LGC Standards, Molsheim, France) and stably expressed the luciferase protein as previously described [12]. MDA-MB-231 and MCF-7 cells were grown in Dulbecco’s modified Eagle’s media (DMEM, Lonza Bioscience, Levallois﻿-Perret, France) containing 10% fetal bovine serum (FBS, Life Technologies, Saint-Aubin, France) in a humidified incubator kept at 37 °C with 5% CO_2_. Stably transfected cells were obtained by transfection of GFP, human apoA-I, and human apoE cDNA-containing plasmids (pReceiver-M68; GeneCopoeia, Inc., Rockville, MD) using EndoFectin (GeneCopoeia, Inc.). Cells were selected with 1.5 μg/ml puromycin.

### Proliferation assays

Proliferation studies were performed using the xCELLigence RTCA DP system (ACEA Biosciences, San Diego, CA). Briefly, after background determination following the manufacturer’s instructions, cells were seeded at 2000 cells/well for both MCF-7 and MDA-MB-231 cells. The impedance value of each well was automatically monitored by the xCELLigence system for a duration of 96 h and expressed as the cell index value. The rate of cell growth was determined by calculating the slope of the line between two given time points.

Proliferation was also determined manually by counting the cells. Cells were seeded in 96-well plates in the media indicated for each experimental condition. At the end of the incubation, culture media were removed and cells were washed and trypsinized. Cells were counted using a hemocytometer.

### Migration assays

For Transwell migration studies, experiments were performed using the xCELLigence RTCA DP system (ACEA Biosciences). Briefly, media in the bottom well contained DMEM with 5% FBS. After background determination, serum-starved cells (in media containing 1% FBS) were added to the top well at 50,000 and 10,000 cells/well for MCF-7 and MDA-MB-231 cells, respectively. Migration was monitored via real-time impedance measurements for 24 h. Experiments were performed according to the manufacturer’s instructions.

### Sterol content determination

Cells were grown to confluence in T175 flasks in complete media (DMEM, 10% FBS). After three washes with PBS, cells were scraped, collected, and centrifuged for 5 min at 200 g. An aliquot was kept for protein quantification, and the remainder was used for lipid quantification. Protein concentration was determined using the bicinchoninic acid assay (Fisher Scientific, Illkirch-Graffenstaden, France) as per the manufacturer’s instructions. For lipid extractions, lipids from the cell pellets were extracted using the method of Bligh and Dyer [13]. Free and esterified cholesterol quantification was performed by HPTLC as previously described [14]. Cellular cholesterol levels were calculated by dividing the cholesterol content by the total protein content.

Sterol and oxysterol quantifications were performed by gas chromatography (GC). After cellular lipid extraction using the method of Bligh and Dyer [13] followed by lipid saponification, alcohol groups were acetylated by hot ethanoyl chloride to allow sterol analysis on a GC ZB5 column (internal diameter, 0.25 mm; film thickness, 0.30 μm; length, 30 m; Phenomenex, Le Pecq, France). Sterol standards (cholesterol, desmosterol, 22-hydroxycholesterol, 24-hydroxycholesterol, 27-hydroxycholesterol) and phytosterols (beta-sitosterol, campesterol, stigmasterol) were acetylated and used as reference standards. Identity confirmation of the peak in the chromatogram was performed by mass spectrometry.

### Fluorescence microscopy

For epifluorescence microscopy imaging, cells were seeded for 24 h on coverslips and then fixed with a 2% solution of formaldehyde (in phosphate-buffered saline containing calcium and magnesium, PBS/CM, Fisher Scientific) for 30 min. After washing with PBS/CM, cells were permeabilized with a 0.1% Triton X-100 solution in PBS/CM/0.2% BSA (solution A) for 10 min. Cells were then incubated in the presence of 50 mM NH_4_Cl in PBS/CM for 10 min to reduce autofluorescence. Detection of apoA-I (antibody 5F6) or apoE (antibody 1D7) was carried out in solution A. After several washes with solution A, cells were incubated in the presence of a secondary antibody (Fragment Rhodamine Red-X-AffiniPure F(ab’)_2_), Jackson ImmunoResearch, Cambridgeshire, UK). After several washes with solution A followed by PBS/CM, the coverslips were sealed on microscope slides in the presence of SlowFade Diamond Antifade containing DAPI (Fischer Scientific). For filipin staining, after fixation, cells were incubated with 0.05 mg/ml filipin (Sigma-Aldrich, Saint-Quentin Fallavier, France) in PBS/CM for 30 min. After several washes with solution A followed by PBS/CM, coverslips were sealed on microscope slides in the presence of Slowfade diamond antifade (Fischer Scientific). Cells were observed on a Nikon TI-S microscope and analyzed with the NIS-BR software (Nikon, France).

For confocal microscopy imaging, cells were seeded for 24 h on coverslips and then fixed with a 2% formaldehyde solution in DPBS/CM for 30 min. After washing with PBS/CM, cells were permeabilized with solution A for 10 min. Cells were then incubated in the presence of 50 mM NH_4_Cl in PBS/CM for 10 min to reduce autofluorescence. Detection of caveolin-1 (antibody N20) was carried out in solution A. After several washes with solution A, cells were incubated in the presence of a secondary antibody (Fragment Rhodamine Red-X-AffiniPure F(ab’)_2_), Jackson ImmunoResearch, Cambridgeshire, UK). After several washes with solution A followed by PBS/CM, the coverslips were sealed on microscope slides in the presence of SlowFade Diamond Antifade containing DAPI (Fischer Scientific). Cells were observed on a Leica SP8 gSTED (Leica) High Resolution Laser Scanning confocal microscope using the LAS-X v2.0 software (Leica, France).

### Cellular mRNA quantification by qPCR

Total cellular mRNAs were purified with the NucleoSpin® RNA Plus kit, according to the protocol established by the manufacturer (Macherey-Nagel, Hoerdt, France). After quantification, mRNAs were reverse transcribed using an RT kit, according to the manufacturer’s instructions (Takara Bio Europe, Saint-Germain-en-Laye, France) using a LabCycler 48 Thermocycler (SensoQuest GmbH, Göttingen, Germany). cDNA were kept at − 80 °C. To quantify mRNAs, a qPCR was performed using the TB Green™ Premix Ex TaqTM kit (Takara Bio Europe) according to the manufacturer’s instructions. Reactions were performed in Hard-Shell® 96-well low-profile skirted PCR plates (Bio-Rad, Marnes-la-Coquette, France) using a CFX Connect™ thermocycler (Bio-Rad) at a Tm of 60 °C. Relative quantification was obtained by calculating the ratio between the values obtained for each gene of interest and the reference gene (GAPDH). Melting curves were routinely performed to determine the specificity of the qPCR reaction. The 2^−ΔΔCt^ method was used for the analysis [15]. The sequences of the primers used are indicated in Table 1.

**Table 1:** Primers used for the qPCR and their sequence

Gene (protein)	Forward primer	Reverse primer
*ABCA1* (ABCA1)	ACCCACCCTATGAACAACATGA	GAGTCGGGTAACGGAAACAGG
*ABCG1* (ABCG1)	CAGGAAGATTAGACACTGTGG	GAAAGGGGAATGGAGAGAAGA
*APOA1* (ApoA-I)	AGCTTGCTGAAGGTGGAGGT	ATCGAGTGAAGGACCTGGC
*APOE* (ApoE)	GGTCGCTTTTGGGATTACCT	CATGGTCTCGTCCATCAGC
*CAV1* (caveolin-1)	ACCCACTCTTTGAAGCTGTTG	GAACTTGAAATTGGCACCAGG
*CDH1* (E-cadherin)	TACGCCTGGGACTCCACCTA	CCAGAAACGGAGGCCTGAT
*FN1* (fibronectin)	CATCGAGCGGATCTGGCCC	GCAGCTGACTCCGTTGCCCA
*GAPDH* (GAPDH)	TGGTCTCCTCTGACTTCAACA	AGCCAAATTCGTTGTCATACC
*HMGCR* (HMGCR)	GTTCGGTGGCCTCTAGTGAG	GCATTCGAAAAAGTCTTGACAAC
*LDLR* (LDLR)	GATAGTGACAATGTCTCACCAAG	CCTCACGCTACTGGGCTTC
*CDH2* (N-cadherin)	GGCGTTATGTGTGTATCTTCACTG	GCAGGCTCACTGCTCTCATA
*SNAIL2* (SNAIL2)	AGACCCTGGTTGCTTCAAGGA	CTCAGATTTGACCTGTCTGCAAA
*SCARB1* (SR-BI)	CGGCTCGGAGAGCGACTAC	GGGCTTATTCTCCATGATCACC
*VIM* (vimentin)	GGCTCGTCACCTTCGTGAAT	GAGAAATCCTGCTCTCCTCGC
*VLDLR* (VLDLR)	GGAGAAGATGAAGAAAACTGTGG	CATCCTGGCCATTGCATAC
*ZEB1* (ZEB1)	GAAAATGAGCAAAACCATGATCCT	CCCTGCCTCTGGTCCTCTTC

### Determination of cellular membrane fluidity modifications

Confluent cells were mechanically detached by flushing with PBS. A suspension of 500,000 cells/ml in PBS was incubated for 15 min at 37 °C with 5 μM of di-4-ANEPPDHQ (amino-naphthylethenylpyridinium (ANEP) probe containing a quaternary ammonium headgroup (DHQ) and a dipropyl) dye probe (Sigma-Aldrich). Excitation of di-4-ANEPPDHQ was performed at 488 nm, and fluorescence emission was collected between 500 and 700 nm (Flexstation 3, Molecular Device, Wokingham, UK). The generalized polarization (GP) value was determined as follows: GP = (*I*_560_ − *I*_650_)/(*I*_560_ + *I*_650_), where *I*_560_ and *I*_650_ represent the fluorescence intensities (areas) at 560 and 650 nm, respectively.

### Animal studies

All mice were housed and maintained at the University of Tours animal facility (medical campus). Mice used in this study were athymic nude mice obtained from Janvier Labs (Le Genest-Saint-Isle, France). Animal protocols used for these studies were approved by the Val de Loire Animal Ethics Committee. MCF-7 cells (5 × 10^6^) were orthotopically injected into the mammary fat pad of 6–8 week-old athymic nude mice implanted the previous day with slow-release 17β-estradiol pellets (0.36 mg/pellet, 60 days; Innovative Research of America, Sarasota, FL). To follow tumor growth, mice were imaged once a week. For each imaging session, mice received a single intraperitoneal injection of luciferin at 150 mg/kg and were held conscious for 10 min post-injection. Mice were anesthetized by isoflurane (~ 3% via inhalation) before the imaging studies. Animals were then transferred to a Caliper IVIS Lumina imager (PerkinElmer, Courtaboeuf, France) for bioimaging.

### Molecular subtype association and survival analysis

Gene expression (DNA microarray data) correlation targeted analysis was applied to published genomic data for patients classified in the same molecular subtype [16, 17]. Basal-like and luminal A subtypes were examined. For these studies, we used GenExMiner (accessed November 2019), as previously described [18, 19].

### Statistical analyses

All values were expressed as the mean ± standard deviation (SD). The Prism 7.0 program (GraphPad Software, Inc., San Diego, CA) was used for statistical analysis. Statistical significance was examined using Student’s *t* test or ANOVA when appropriate (if not, the non-parametric equivalents). Unless otherwise indicated, results are representative of three independent experiments. For patient survival studies, a subgroup analysis was performed according to the ER status, or based on molecular subtypes, by single sample predictors (SSPs) subtyping method. The prognostic impact of *APOA-I* and *APOE* genes was evaluated using univariate Cox proportional hazards model and illustrated with a Kaplan-Meier curve.

## Results

### ApoA-I and ApoE expression regulate cellular cholesterol distribution in MCF-7 and MDA-MB-231

**Fig. 1:**
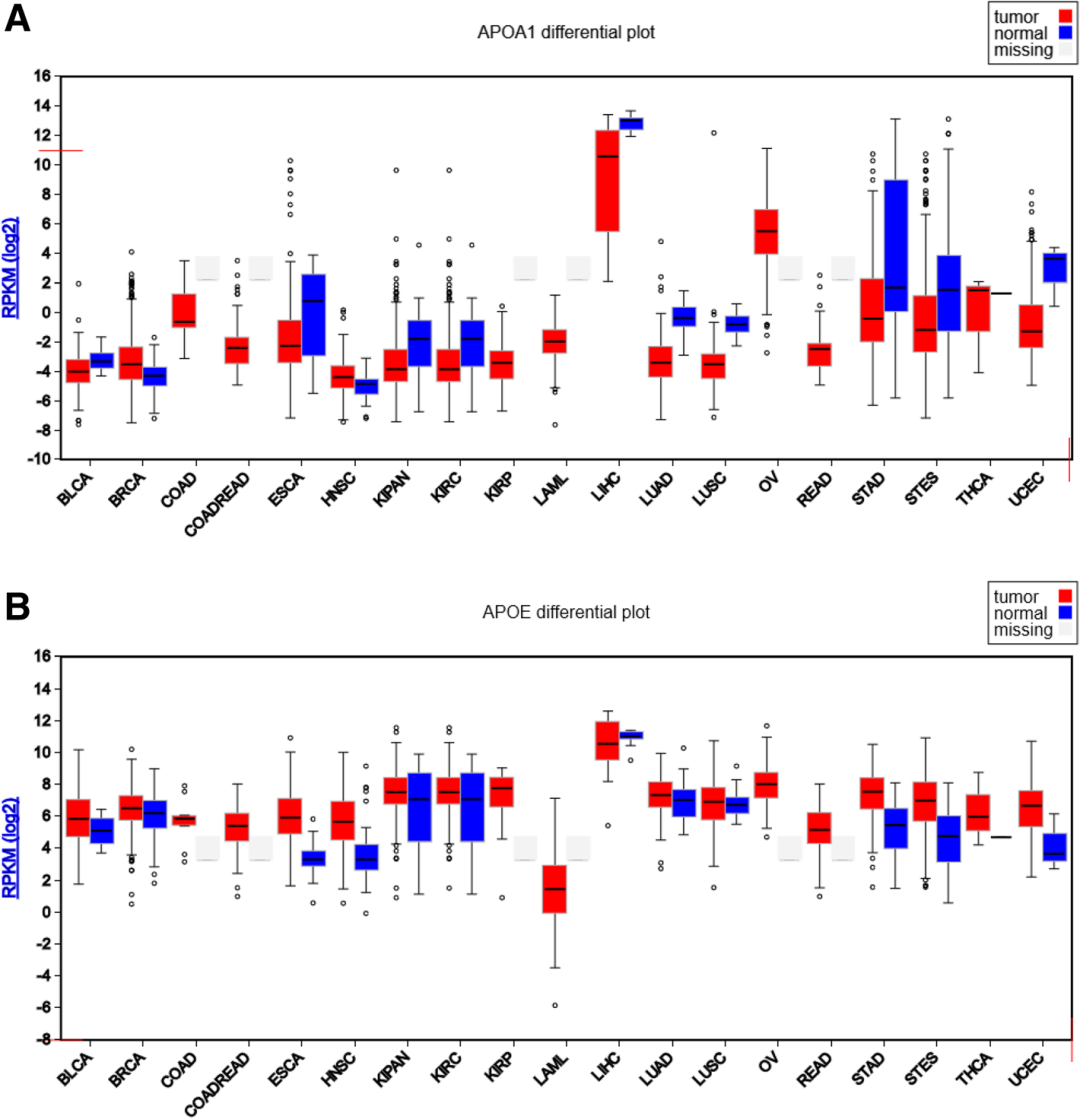
mRNA levels in various types of tumors and their corresponding normal tissue. The RPKM mRNASeq expression profiles are presented for each TCGA disease chart. The results are, in part, based upon the data generated by the TCGA Research Network: https://www.cancer.gov/tcga. This graphical presentation was obtained using the FireBrowse tool. The results for the following tissues (*n* = tumor samples; normal samples) are presented: BLCA, bladder urothelial carcinoma (*n* = 49; 11); BRCA, breast invasive carcinoma (*n* = 699; 88); COAD, colon adenocarcinoma (*n* = 10); COADREAD, colorectal adenocarcinoma (*n* = 75); ESCA, esophageal carcinoma (*n* = 185; 13); HNSC, head and neck squamous cell carcinoma (*n* = 236; 27); KIPAN, pan-kidney cohort (KICH+KIRC+KIRP) (*n* = 446; 68); KIRC, kidney renal clear cell carcinoma (*n* = 433; 68); KIRP, kidney renal papillary cell carcinoma (*n* = 13); LAML, acute myeloid leukemia (*n* = 176); LIHC, liver hepatocellular carcinoma (*n* = 17; 9); LUAD, lung adenocarcinoma (*n* = 120; 37); LUSC, lung squamous cell carcinoma (*n* = 212; 17); OV, ovarian serous cystadenocarcinoma (*n* = 299); READ, rectum adenocarcinoma (*n* = 65); STAD, stomach adenocarcinoma (*n* = 272; 33); STES, stomach and esophageal carcinoma (*n* = 457; 46); THCA, thyroid carcinoma (*n* = 3); UCEC, uterine corpus endometrial carcinoma (*n* = 259; 3). A gray box was drawn to indicate that no normal samples were available for that disease cohort. **a** mRNASeq profiles obtained for *apoA1*. **b** mRNASeq profiles obtained for *apoE*. RPKM, reads per kilobase million values are indicated

Results presented in Fig. 1 are based upon the data generated by the TCGA Research Network [20]. A graphical presentation was obtained using the FireBrowse tool. Figure 1 shows that *APOA1* was barely detectable in tumors obtained from breast cancer patients and most other tumor types and the corresponding healthy tissues. Only liver tumors and normal livers obtained from human patients displayed significant levels of *APOA1* mRNA (Fig. 1a). Expression levels of *APOE*, although more elevated than those of *APOA1*, also remained very low, and similar in healthy and cancerous tissues, except for the liver again (Fig. 1b).

**Fig. 2:**
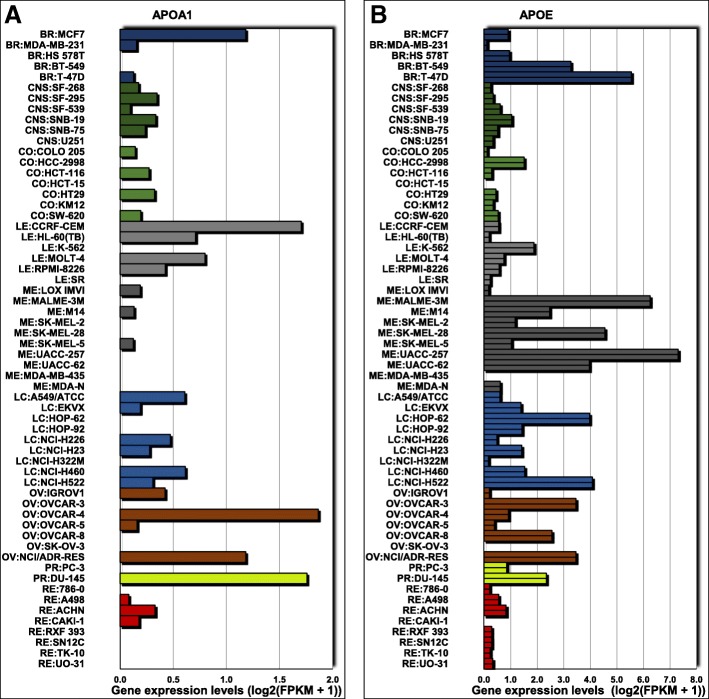
mRNA levels of *APOA1* (**a**) and *APOE* (**b**) in a panel of 60 diverse human cancer cell lines (NCI-60) used by the Developmental Therapeutics Program of the US National Cancer Institute. mRNA levels were obtained via the CellMiner™ web application available at https://discover.nci.nih.gov/cellminer/home.do [21, 22]

In a panel of 60 diverse human cancer cell lines (NCI-60) used by the Developmental Therapeutics Program of the US National Cancer Institute, we also found that both *APOA1* (Fig. 2a) and *APOE* (Fig. 2b) mRNA levels were low [21, 22], with the exception, for apoE only, of the T-47D cell line and melanoma cell lines. Therefore, to modulate cellular cholesterol metabolism in MCF-7 and MDA-MB-231 cells, we expressed apoA-I and apoE. These cells were transfected with GFP (control), human apoA-I, or human apoE cDNA-containing plasmids. Transfected cells were then selected and amplified. The expression of apoA-I or apoE was verified by qPCR (Suppl. Figure 1a) and immunofluorescence (Suppl. Figure 1b,c).

**Fig. 3:**
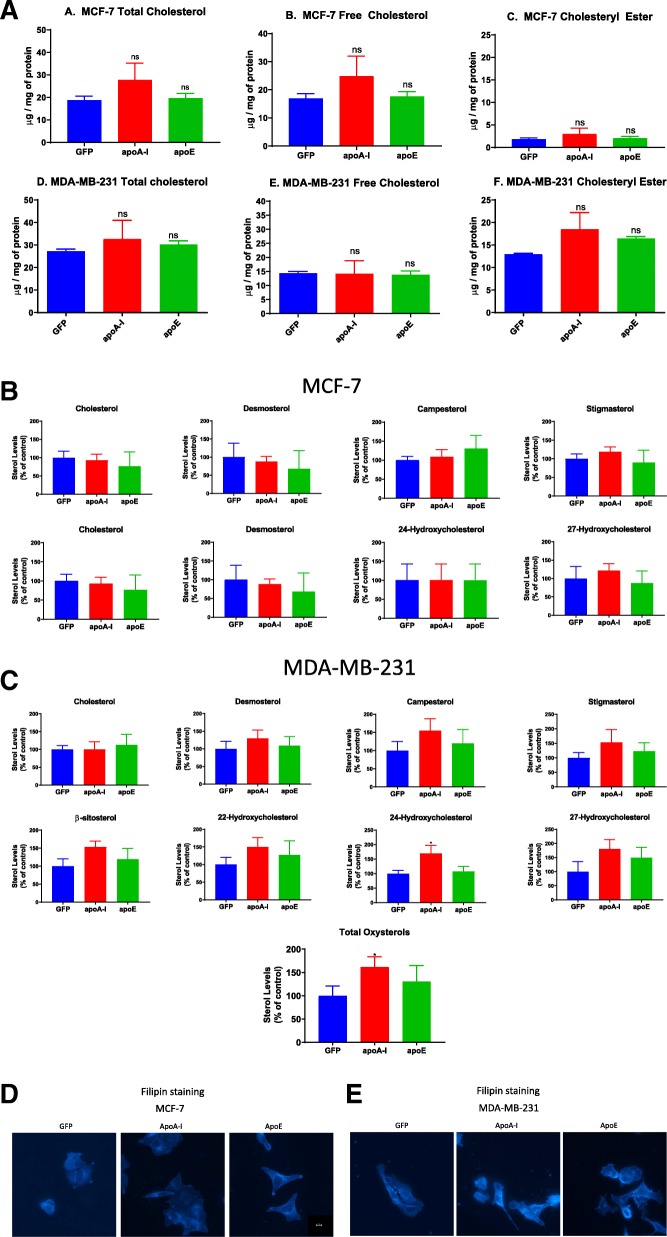
ApoA-I and apoE expression alter cholesterol metabolism in transfected MCF-7 and MDA-MB-231 cells. **a** Measurement of cellular cholesterol content (total, free, esterified) by HPTLC in transfected MCF-7 and MDA-MB-231 cells. Values represent means ± SD. ns, difference with control not significant. Significant difference compared to control cells: *P < 0.05. Measurement of sterol content by gas chromatography (GC) in transfected MCF-7 (**b**) and MDA-MB-231 (**c**) cells . After lipid extraction and saponification of the cellular extracts, cholesterol, desmosterol, 27-hydroxycholesterol, 22-hydroxycholesterol, 24-hydroxycholesterol, 27-hydroxycholesterol, beta-sitosterol, campesterol, and stigmasterol were quantified by GC. Values are means (± SD). Significant difference compared to control cells: *P < 0.05. **d**, **e** Visualization of free cholesterol by filipin staining in transfected MCF-7 and MDA-MB-231 cells. Free cholesterol was detected by fluorescence microscopy (DAPI channel) after cell incubation with filipin

We first examined cholesterol levels in both cell lines. In both cases, we found that neither apoA-I nor apoE expression remarkably affected esterified or free cholesterol levels in MCF-7 cells (Fig. 3a). In MDA-MB-231, apoA-I was responsible for a marginally significant increase in esterified cholesterol (*P* = 0.0507) (Fig. 3a).

We also examined whether other sterol (cholesterol, desmosterol, 22-hydroxycholesterol, 24-hydroxycholesterol, 27-hydroxycholesterol) levels were affected in these cell lines. These sterols have previously been shown to affect cholesterol metabolism and cellular properties of cancer cells [23]. While no changes in the different sterol levels were observed in MCF-7 cells (Fig. 3b), apoA-I expression increased 24-hydroxycholesterol and total oxysterol levels in MDA-MB-231 cells (Fig. 3c). Given the possibility that phytosterols (e.g., campesterol, stigmasterol, β-sitosterol) may play a role in cancer progression [24], we examined their levels in cell extracts. However, no significant differences were observed between the cell lines (Fig. 3c).

When cellular free cholesterol distribution was examined by fluorescence microscopy using the filipin probe, differences were observed. In MCF-7 expressing apoA-I or apoE, the intracellular free cholesterol content was greater than in the MCF-7 control cells (Fig. 3d). On the other hand, in MDA-MB-231 expressing apoA-I or apoE, free cholesterol association with the plasma membrane was enhanced compared to the GFP-expressing cell line (Fig. 3e).

### Determination of cellular membrane fluidity changes

**Fig. 4:**
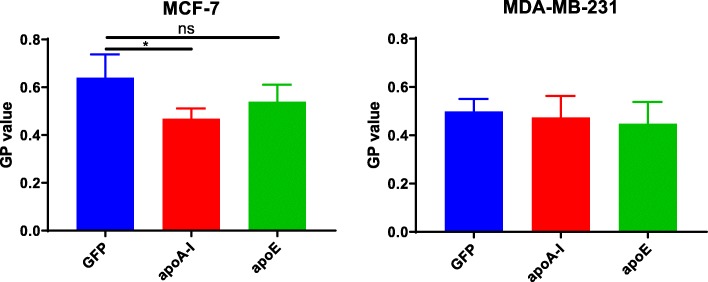
Membrane fluidity measurement. **a** Membrane fluidity measurement in MCF-7 cells. **b** Membrane fluidity measurement in MDA-MB-231 cells. Measurements of membrane fluidity were performed using the Di-4-ANEPPDHQ probe. Compared with a control condition, an increase in the GP value means that the plasma membrane is more rigid, whereas a decrease in the GP value means that the plasma membrane is more fluid. ns, difference with control not significant. Significant difference compared to control cells: *P < 0.05

Since the cholesterol content of biological membranes is an important regulator of membrane fluidity [25], we examined whether apolipoprotein expression could affect membrane fluidity in the different cell lines. In MCF-7 cells, measurements of membrane fluidity using the di-4-ANEPPDHQ probe indicated that apoA-I expression could significantly increase membrane fluidity in this context (Fig. 4). A similar but not significant trend was observed for apoE in MCF-7 cells. However, no effect was observed in MDA-MB-231 cells (Fig. 4).

### ApoA-I and apoE increase proliferation of MCF-7 cells but reduce that of MDA-MB-231 cells

**Fig. 5:**
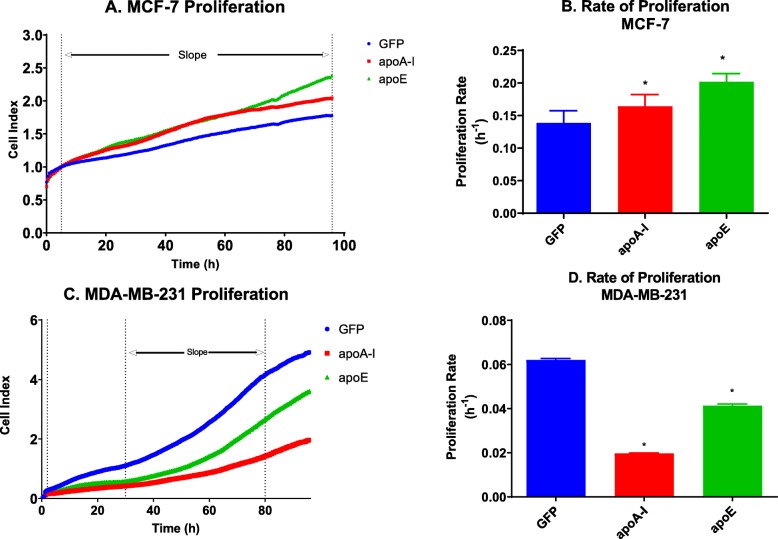
ApoA-I and apoE increase the proliferation of MCF-7 but not of MDA-MB-231 cells. **a** Proliferation curve of the different MCF-7 cell lines. **b** Proliferation rate of the different MCF-7 cell lines. **c** Proliferation curve of the different MDA-MB-231 cell lines. **d** Proliferation rate of the different MDA-MB-231 cell lines. Proliferation was measured by impedance measurements (xCELLigence) in real-time (**a**, **c**), and proliferation rates were determined between the dashed lines for each cell line (**c**, **d**). ns, difference with control not significant. Significant difference compared to control cells: *P < 0.05

To determine the impact of apoA-I and apoE expression on cancer cells, proliferation of the transfected cells was examined using the xCELLigence RTCA DP system (ACEA Biosciences). Measuring proliferation under these conditions, we observed that MCF-7 cells expressing apoA-I or apoE proliferated faster than control cells. MCF-7 expressing apoE displayed the most elevated proliferation rate. These results therefore indicate that apoA-I and apoE stimulate the proliferation of MCF-7 cells (Fig. 5a, b). Very similar results were observed when proliferation was measured by counting cells manually (suppl Figure 2a). For MDA-MB-231, the reverse observations were made. The expression of either apoA-I or apoE in MDA-MB-231 was associated with a reduction in cellular proliferation compared to control cells. In that case, apoA-I expression was the most effective at decreasing the proliferation of MDA-MB-231 cells (Fig. 5c, d). Very similar results were observed when proliferation was measured by counting cells manually (suppl Figure 2b).

### ApoA-I and apoE increase migration of MCF-7 cells but decrease that of MDA-MB-231 cells

**Fig. 6:**
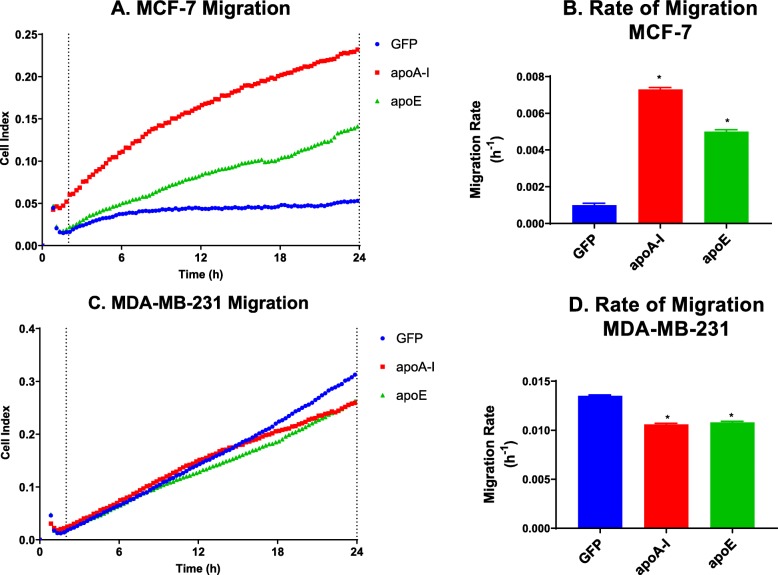
ApoA-I and apoE increase the migration of MCF-7 cells but decrease that of MDA-MB-231 cells. **a** Migration curve of the different MCF-7 cell lines. **b** Migration rate of the different MCF-7 cell lines. **c** Migration curve of the different MDA-MB-231 cell lines. **d** Migration rate of the different MDA-MB-231 cell lines. Migration was obtained by creating a gradient of 1 (top well) to 10% (bottom well) serum. Migration was measured by impedance measurements (xCELLigence) in real-time (**a**, **c**), and migration rate was then determined between the dashed lines for each cell line (**c**, **d**). Significant difference compared to control cells: *P < 0.05

Transwell assays and wound healing studies were realized to test the effect of apoA-I and apoE on the regulation of cellular migration. For MCF-7 cells, despite their intrinsic reduced ability to migrate, the expression of either apoA-I or apoE was associated with a remarkable increase in the migration of MCF-7 cells (Fig. 6a, b) in Transwell assays. However, either apoA-I or apoE expression could reduce MDA-MB-231 cellular migration in Transwell assays (Fig. 6c, d).

### ApoA-I and apoE prime MCF-7 cells for epithelial-to-mesenchymal transition

**Fig. 7:**
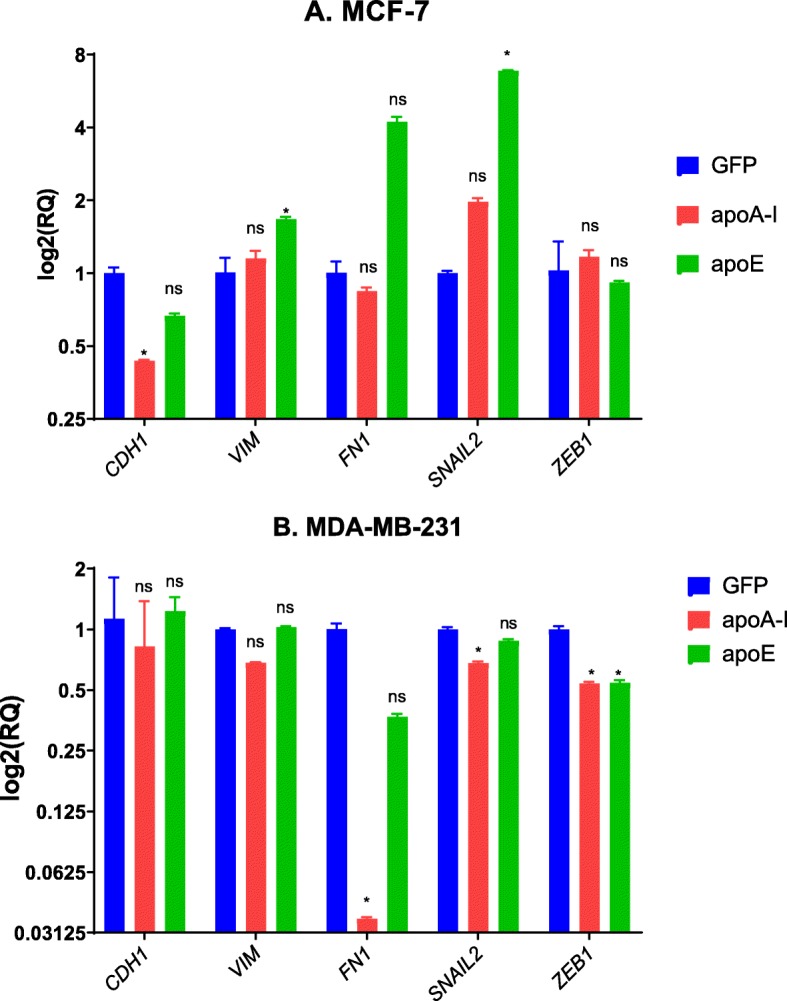
Expression of EMT markers in MCF-7 and MDA-MB-231 cells. EMT marker mRNA levels were determined after mRNA isolation from 90% confluent cell cultures. mRNA levels of *CDH1*, *VIM*, *FN1*, *SNAIL2*, and *ZEB1* were quantified by RT-qPCR. Values are means (± SD). **a** Expression of EMT markers in MCF-7 cells. **b** Expression of EMT markers in MDA-MB-231 cells. ns, difference with control not significant. Significant difference compared to control cells: *P < 0.05

Cancer progression depends on the epithelial-to-mesenchymal transition (EMT) process, which allows the de-differentiation of epithelial cells to a mesenchymal-like phenotype and increases cancer cell proliferation and migration [26]. While the MCF-7 cell line exhibits an epithelial phenotype, the MDA-MB-231 cell line already has a mesenchymal phenotype. Induction of EMT in MCF-7 cells may therefore be responsible for the increased proliferative and migratory potential of the cells expressing apoA-I and apoE. To verify the state of the EMT in our cell lines, we analyzed by qPCR the mRNA levels of key genes involved in the EMT process (Fig. 7). At the mRNA level, a decrease in *CDH1* (encoding E-cadherin) expression in MCF-7 cells expressing apoA-I was significant (Fig. 7a). With apoE, a trend toward a decrease was observed. In MCF-7 cells expressing apoA-I, there were also no significant changes in the expression of *FN1* (encoding fibronectin, a marker of extracellular matrix adhesion), *VIM* (encoding vimentin, a mesenchymal marker), or *ZEB1* (encoding an EMT marker). *SNAIL2* (encoding an EMT marker) mRNA levels were marginally significantly increased. In MCF-7 cells expressing apoE, there was a marginally significant increase in *FN1*, an increase in the expression of *VIM* and *SNAIL2*, but no change in *ZEB1* expression was observed. Taken together, these results suggest a stimulation of the EMT process in MCF-7 cells expressing either apoA-1 or apoE.

In MDA-MB-231 cells, we observed alterations supporting the idea that either apoA-I or apoE inhibits the EMT (Fig. 7b). Accordingly, we found that MDA-MB-231 cells expressing apoA-I displayed reduced levels of *SNAIL2*, *ZEB1*, and *FN1*. No change in the expression of *CDH1* was observed. In MDA-MB-231 cells expressing apoE, if no changes in the expression of *CDH1* or *VIM* were observed, *SNAIL2* and *ZEB1* mRNA levels were found to be significantly decreased, and *FN1* mRNA levels were marginally significantly decreased.

### Regulation of EMT induced by TGFβ in MCF-7 cells

**Fig. 8:**
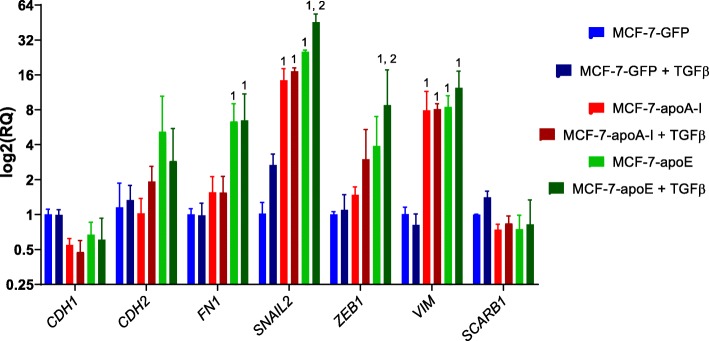
TGFβ signaling promotes the EMT in MCF-7 cells. EMT marker mRNA levels were determined after mRNA isolation from MCF-7 cells grown in the presence of TGFβ for 24 h. mRNAs were isolated from cells incubated under control conditions (ctrl, no TGFβ) or with TGFβ (+TGFβ) in complete medium for 24 h. mRNA levels of *CDH1*, *CDH2*, *FN1*, *SNAIL2*, *ZEB1*, *VIM*, and *SCARB1* were quantified by RT-qPCR. Values are means (± SD). ^1^Significant difference compared to control cells: *P < 0.05. ^2^Significant difference compared to non-TGFβ-treated cells: *P < 0.05

TGFβ is a well-known inducer of EMT [27]. To determine the effect of TGFβ on EMT in MCF-7 cells, we incubated these cell lines with TGFβ for 24 h and examined by qPCR the transcription levels of key genes involved in the EMT process (Fig. 8). As previously observed, *SNAIL2* levels were increased by apoA-I and apoE expression, and cellular incubation with TGFβ was associated with a further increased of *SNAIL2* levels in apoE-expressing cells. Importantly, *ZEB1* was also induced by TGFβ in apoE-expressing cells. Taken together, these data suggest that apoA-I and apoE can act as activators of the EMT process in MCF-7 cells.

### Expression modulation of genes implicated in the regulation of cholesterol metabolism

**Fig. 9:**
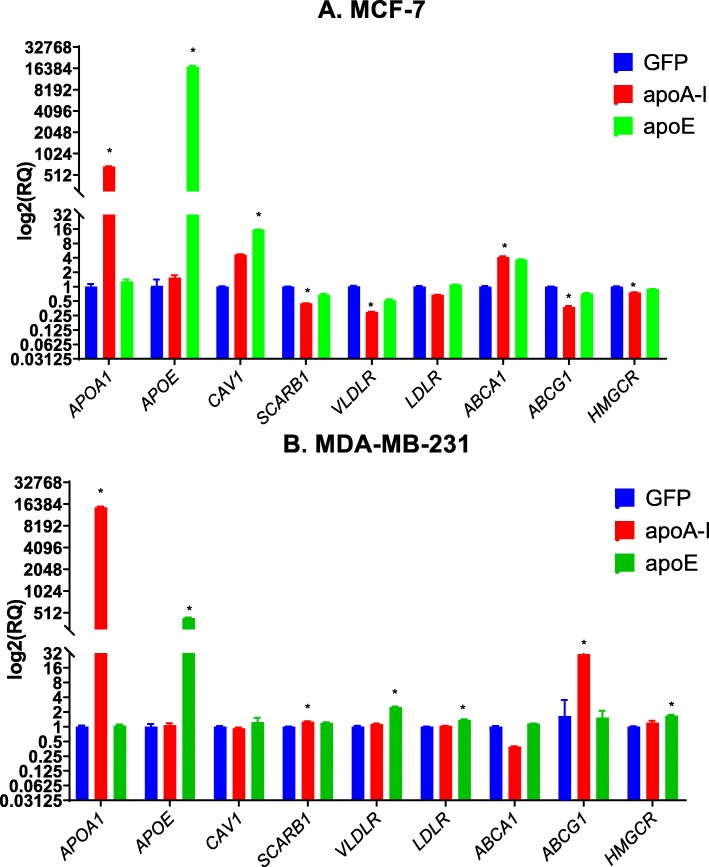
Alterations in the regulation of cholesterol regulatory response. Cholesterol regulatory gene product mRNA levels were determined after mRNA isolation from 90% confluent cell cultures. mRNA levels of *APOA1*, *APOE*, *CAV1*, *SCARB1*, *VLDLR*, *LDLR*, *ABCA1*, *ABCG1*, and *HMGCR* were quantified in MCF-7 cells (**a**) and MDA-MB-231 cells (**b**). Significant difference compared to control cells: *P < 0.05.

To determine if the observed effects could be associated with alterations in the modulation of cholesterol metabolism-regulating genes, we examined, at the mRNA level, their expression (Fig. 9). In MCF-7 cells expressing apoA-I (Fig. 9a), *CAV1* mRNA levels were marginally significantly increased, and ABCA1 mRNA levels were significantly increased. On the other hand, *SCARB1* (encoding SR-BI), *VLDLR*, *HMGCR*, and *ABCG1* mRNA levels were reduced. In MCF-7 cells expressing apoE, *ABCA1* mRNA levels were marginally significantly increased, and *CAV1* levels were significantly increased. However, levels of *VLDLR*, *SCARB1*, *ABCG1*, and *HMGCR* were not affected (Fig. 9a).

In MDA-MB-231 cells expressing apoA-I (Fig. 9b), *SCARB1* mRNA levels were slightly increased. Expression levels of *ABCG1* were remarkably increased. In apoE-expressing cells, *CAV1*, *SCARB1*, and *ABCG1* levels remained unchanged. However, the expression levels of *VLDLR*, *LDLR*, and *HMGCR* were increased (Fig. 9a). Regarding *CAV1*, while no differences in the expression levels were observed at the mRNA level, confocal microscopy of MDA-MB-231 cells shows an increased plasma membrane localization of the corresponding protein (Suppl. Figure 3). Low expression levels of caveolin-1 in MCF-7 did not allow us to detect this protein under the same conditions.

Taken together, these data suggest that apoA-I or apoE expression can induce major changes in the regulation of cholesterol metabolism-regulating genes.

### Effect of apoA-I and apoE on tumor formation in vivo

**Fig. 10:**
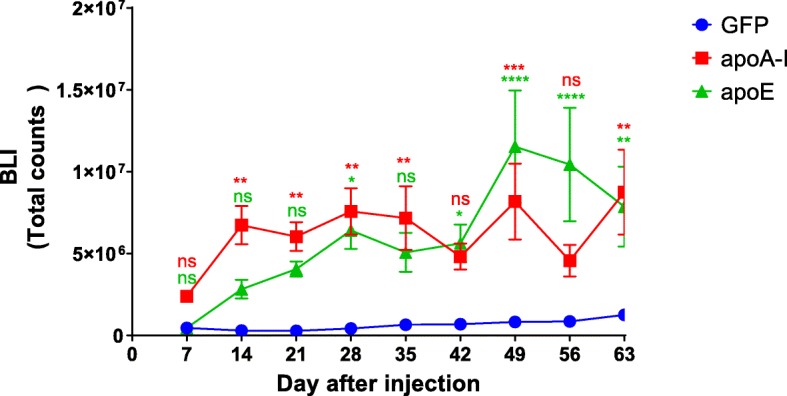
Effect of apoA-I and apoE on tumor formation in vivo. MCF7 cells (5 × 10^6^) were orthotopically injected into the mammary gland fat pad of 9-week-old athymic nude mice implanted with slow-release 17β-estradiol pellets (0.36 mg/pellet, 60 days). Tumor growth was followed for the next 60 days by bioluminescence (BLI). BLI was used to follow tumor growth after luciferin injection; mice were imaged once a week. For each imaging session, mice received a single intraperitoneal injection of luciferin at 150 mg/kg and were held conscious for 10 min post-injection (*n* = 10 for MCF-7-GFP and MCF-7-apoE, *n* = 12 for MCF-7-apoA-I). Significant differences compared to control cells: *P < 0.05; **P < 0.01; ***P < 0.001; ****P < 0.0001

To determine the role of apoA-I and apoE in tumor growth of MCF-7 cells, the different MCF-7 cell lines were orthotopically injected into the mammary fat pad of athymic nude mice after implantation with slow-release 17β-estradiol pellets. Tumor growth was followed by bioluminescence analysis for 60 days after injection. Tumor growth curves presented Fig. 10 indicate that, as predicted by in vitro experiments, tumor growth was significantly enhanced with MCF-7 expressing apoA-I or apoE compared to GFP-expressing cells.

## Discussion

Our study demonstrates for the first time that apoA-I and apoE expression in two different types of human breast cancer cell lines can have very different consequences on the cancer properties of these cell lines. We observed that apoA-I and apoE increase the aggressive potential of a luminal A model but decrease the aggressiveness of a triple-negative model.

In the present study, we have shown that apoA-I and apoE can modulate cholesterol distribution in MCF-7 cells and in MDA-MB-231 cells. However, the types of effect that we observed were dependent on the cell line. In MCF-7 cells, both apolipoproteins appear to reduce or limit cholesterol transfer to the plasma membrane. However, in MDA-MB-231 cells, the reverse effect was observed. This important observation combined with the very different effects of apolipoproteins on cellular proliferation and migration potential in the two cell lines suggests that these cell types present with intrinsic cholesterol metabolism differences. In fact, while free cholesterol levels appear to be very similar (Fig. 3a), we observed an increase in cholesteryl ester levels in MDA-MB-231. This finding is consistent with previous studies that have demonstrated increased intratumoral accumulation of cholesteryl ester in aggressive triple-negative tumors [28]. Taken together, these data suggest that modulation of cholesterol metabolism will have a more significant effect in the MDA-MB-231 cell line than in the MCF-7 cell line. Data from the panel of 60 diverse human cancer cell lines (NCI-60) used by the Developmental Therapeutics Program of the US National Cancer Institute also suggest important differences in the expression of two essential proteins involved in the regulation of cholesterol efflux. While *ABCA1* mRNA levels appear to be more elevated in MDA-MB-231 cells than in MCF-7 cells, the reverse is observed for *ABCG1*. This observation is important since ABCA1 expression has been shown to be important for lipid association of apoA-I (and probably apoE) in other cell types such as hepatocytes [29]. On the other hand, ABCG1 is more important for cholesterol efflux to HDL lipoproteins [30]. This finding is important and may explain the increased transfer of cholesterol to the plasma membrane in MDA-MB-231. This transfer may be inefficient in MCF-7 cells, thereby leading to intracellular accumulation of free cholesterol.

Another important difference between MCF-7 and MDA-MB-231 cells is the presence of the estrogen receptor (ER). Interestingly, when ERα is expressed in MDA-MB-231 cells, its activation inhibits cellular proliferation contrary to the increased proliferation observed with MCF-7 cells [31]. These differences may be due to the presence of distinct signaling pathways in the two cell lines. Alternatively, they may also suggest that different cellular cholesterol distribution may differently affect signaling via ERα. In addition, differences in cholesterol metabolism may also be associated with the expression of the enzyme SULT2B1b, which is present in MCF-7 cells but not in MDA-MB-231 [32]. This enzyme is responsible for the sulfation of sterol derivatives including 5,6α-epoxy-cholesterol. Interestingly, inhibition of the cholesterol-5,6-epoxide hydrolase has been shown to induce production of 5,6α-epoxy-cholesterol, which can, when sulfated by SULT2B1b, promote apoptosis [32]. Finally, previous studies have also indicated that the activity of the enzyme acyl:cholesteryl ester transferase (ACAT) and cholesteryl ester availability/production may regulate cellular proliferation and migration [33–35]. Our study suggests that, in MDA-MB-231, a flux of cholesterol toward an exit from the cells is promoted. Nevertheless, we did not observe a significant change in the levels of esterified cholesterol in MDA-MB-231 cells expressing apoA-I or apoE compared to control cells. It is therefore possible that this pool of esterified cholesterol is not as accessible as in the control cells.

Caveolin-1 is a protein that has been extensively characterized [36, 37] and has been shown to play an important role in the regulation/inhibition of various signaling pathways associated with cancer progression [38]. Therefore, in MDA-MB-231, an increased localization of caveolin-1 at the plasma membrane may be associated with reduced signaling via various pathways such as the MAP kinase and PI3 kinase pathways. The latter pathways are important since they are known to regulate the proliferation and migration of MDA-MB-231 cells [9]. Therefore, increased plasma membrane localization of caveolin-1 may inhibit signaling via these pathways and lead to the observed reduced proliferation in our model MDA-MB-231 cells.

In MCF-7 cells, apoA-I and apoE expression was associated with increased proliferation and migration of these cells. Furthermore, we also show that these apolipoproteins facilitate the EMT process. It is possible that increased signaling via TGFβ is involved in this pathway. Our data also suggest that increased caveolin-1 expression may be associated with increased proliferation of this cell line. Contrary to MDA-MB-231 cells, MCF-7 cells express the estrogen receptor (ER), and studies have demonstrated that caveolin-1 could potentiate signalization via ER [39]. The ER signaling pathway may also promote the EMT process, as previously observed [40].

Our data also show important differences in terms of sterol content for these two cell lines. It appears that in MCF-7 cells, most of the sterol population is represented by cholesterol. By contrast, in MDA-MB-231, desmosterol and phytosterols also represent a significant portion of the sterol content. In this study, the presence of phytosterols most likely originated from the incubation with fetal serum, which provided phytosterols to the cells. Importantly, MDA-MDA-231 cells also contain significant levels of oxysterols. We show that 24-hydroxycholesterol and total oxysterol levels are increased in MDA-MB-231 cells expressing apoA-I in comparison with control MDA-MB-231 cells. Importantly, these oxysterols have been shown to act as ligands for the activation of the nuclear receptors LXR [41]. These nuclear receptors play an important role in the regulation of cholesterol metabolism. Specifically, *ABCG1* is an important target gene of LXR, and we show that its mRNA levels are upregulated by apoA-I. In particular, 27- and 24-hydroxycholesterol are known activator of LXRα and LXRβ [41]. However, other pathways are also regulated by these nuclear receptors. They include cell cycle regulatory pathways (decreased Skp2, cyclin A2, cyclin D1, increased p53), which allow a reduction in the cellular proliferation of cancer cells [42]. Synthetic and natural activators have been successfully used to limit cancer progression [42, 43]. Another endogenously produced activator of LXRβ has recently been identified and shown to lead to lethal autophagy in cancer cells [44]. However, studies with 27- and 25-hydroxycholesterol have also shown that these oxysterols can act as selective estrogen receptor modulators. Therefore, depending on the type of oxysterol and the type of tumor, different profiles of sterols may be identified and may have different effects on tumor cells. For MCF-7 cells, it cannot be excluded that local increase in 27- and/or 25-hydroxycholesterol could activate ER in an autocrine manner and promote proliferation and migration of cells expressing apoA-I and apoE.

Our data also show that in MDA-MB-231 cells expressing apoA-I or apoE, we observed important compensatory mechanisms regarding the metabolism of cholesterol. If apoA-I and apoE are effective at promoting cholesterol efflux in the cells, it is expected that these cell lines will upregulate the transcription of genes responsible for the uptake and/or synthesis of cholesterol. In agreement with this hypothesis, we observed increased mRNA levels of *SCARB1*, *VLDLR*, *LDLR*, and *HMGCR* in MDA-MB-231 cells expressing apoA-I or apoE. On the contrary, if cholesterol efflux is inefficient, we should not observe any major change, as in the case of MCF-7 cells. Taken together, these data suggest that apoA-I and apoE can modulate cholesterol metabolism in MDA-MB-231, probably by increasing cholesterol flux. However, in MCF-7, these apolipoproteins cannot induce the same effects and their expression may further limit the cellular flux of cholesterol.

**Fig. 11:**
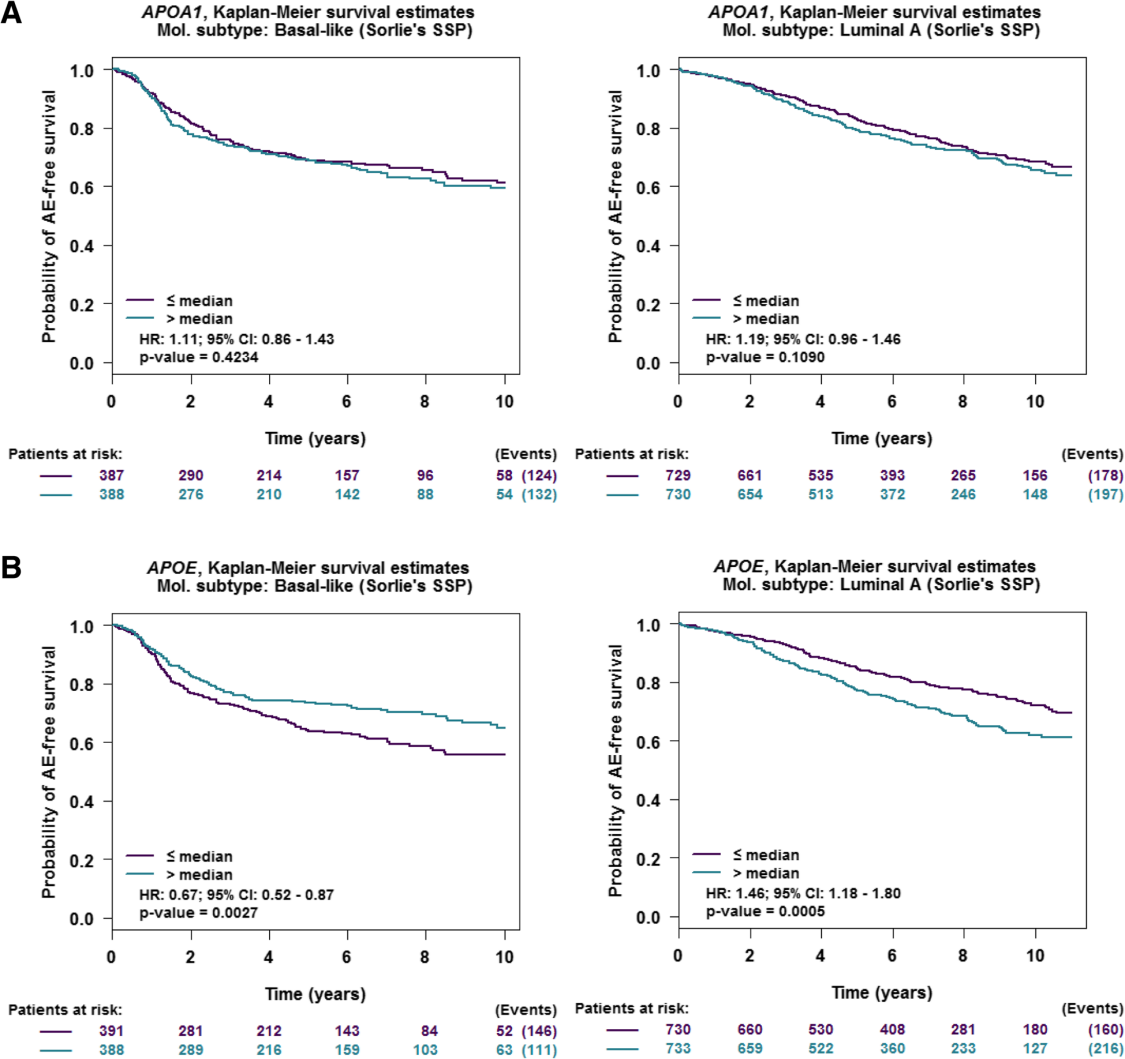
**a** Kaplan-Meier relapse-free survival curves of breast cancer subtypes stratified by low/high *APOA1* gene expression. **b** Kaplan-Meier relapse-free survival curves of breast cancer subtypes stratified by low/high *APOE* gene expression. Gene expression (microarray data) correlation targeted analysis was applied to published genomic data for patients classified in the same molecular subtype [16, 17]. For these studies, we used GenExMiner, as previously described [18, 19]

Interestingly, we have also examined the importance of apoA-I and apoE in patients with different types of tumors (Fig. 11a, b). *APOAI* gene expression does not appear to have any effect on any adverse event (e.g., relapse, metastases) in patients carrying either basal-like or luminal A tumors. By contrast, increased expression levels of *APOE* is associated with improved survival in patients with basal-like tumors (HR = 0.67 [0.52–0.87]) but with reduced survival in patients with luminal A tumors (HR = 1.46 [1.18–1.80]). For apoE, these observations are consistent with the present study in which we show that apoE limits the proliferation and migration of MDA-MB-231 cells but increases the proliferation and migration of MCF-7 cells. For apoA-I, its corresponding gene promoter is found to be highly methylated in many tissues other than the liver [45], and it may therefore be difficult to detect any expression in the mammary tissue as demonstrated in Fig. 1a.

## Conclusions

Our study shows that effective modulation of cholesterol metabolism can limit cancer cell proliferation and migration. Interestingly, modulation of cholesterol metabolism by apoA-I and apoE is very effective in the triple-negative cell line MDA-MBA-231 and may also improve the treatment and survival of patients carrying these types of tumors. 6-Oxo-cholestan-3β,5α-diol has recently been identified as a tumor promoter in ER+ and triple-negative cells [46]. In future studies, it may be interesting to modulate the availability of this sterol via the regulation of cholesterol metabolism and thereby limit tumor progression and reduce resistance to treatment.

## Supplementary information

**Additional file 1:Supplementary Figure 1.** Expression of ApoA-I and ApoE in Transfected MCF-7 and MDA-MB-231 Cells. A. mRNA levels in transfected MCF-7 and MDA-MB-231 cells. mRNA levels of *APOA1* and *APOE* were quantified by RT-qPCR. ns: difference with control not significant. Significant difference compared to control cells: *P <0.05. B and C. Immunofluorescence detection of apoA-I and apoE in transfected MCF-7 and MDA-MB-231 cells. ApoA-I and apoE were detected by immunofluorescence using anti-apoA-I and anti-apoE antibodies and visualized by epifluorescence microscopy (scale = 10 μm).

**Additional file 2:Supplementary Figure 2.** ApoA-I and apoE increase proliferation of MCF-7 (A) but not of MDA-MB-231 (B) cells as determined by cell counting measurements. ns: difference with control not significant. Significant difference compared to control cells: *P <0.05.

**Additional file 3:Supplementary Figure 3.**: Immunostaining of caveolin-1 in MDA-MB-231 cells as evaluated by confocal microscopy.

## Data Availability

All data generated or analyzed during this study are included in this published article.
