# Supplementary figures and images for: Apolipoprotein-mediated regulation of lipid metabolism induces distinctive effects in different types of breast cancer cells
Source: Breast Cancer Res. 2020 Apr 22;22:38. doi: 10.1186/s13058-020-01276-9 (PMC7178965; doi:10.1186/s13058-020-01276-9)

# Suppl. Figure 1A

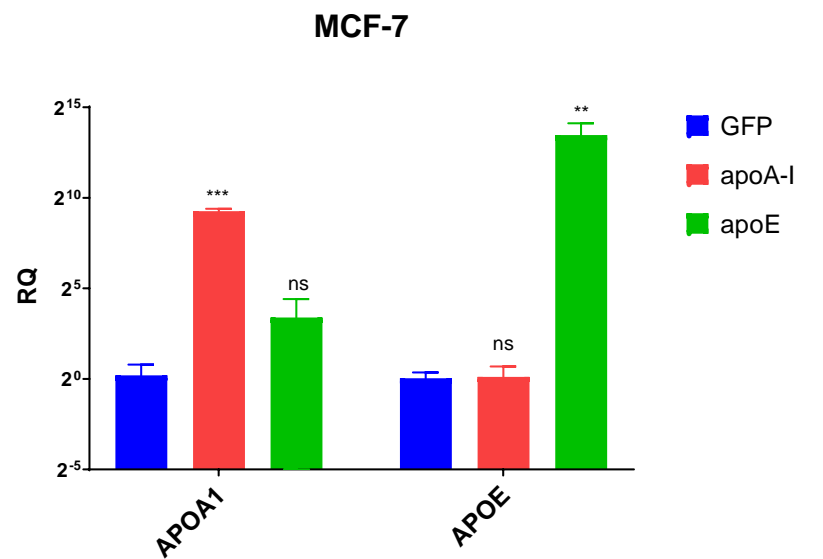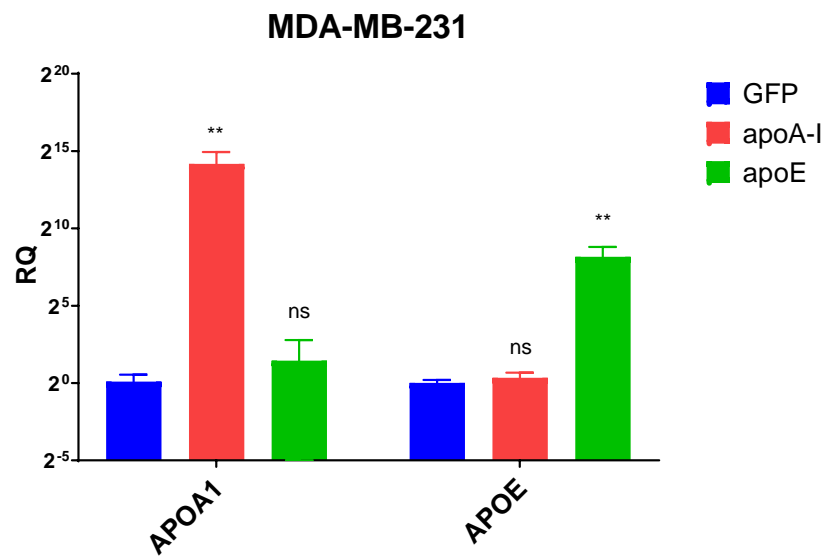

Supplement: Supplementary file 1 — Additional file 1:Supplementary Figure 1. Expression of ApoA-I and ApoE in Transfected MCF-7 and MDA-MB-231 Cells. A. mRNA levels in transfected MCF-7 and MDA-MB-231 cells. mRNA levels of APOA1 and APOE were quantified by RT-qPCR. ns: difference with control not significant. Significant difference compared to control cells: *P <0.05. B and C. Immunofluorescence detection of apoA-I and apoE in transfected MCF-7 and MDA-MB-231 cells. ApoA-I and apoE were detected by immunofluorescence using anti-apoA-I and anti-apoE antibodies and visualized by epifluorescence microscopy (scale = 10 μm). [file 13058_2020_1276_MOESM1_ESM.zip › Suppl Figure 1/Suppl Figure 1A.pdf]

MCF-7

# Suppl. Figure 1B

GFP

ApoA-I

ApoE

ApoA-I

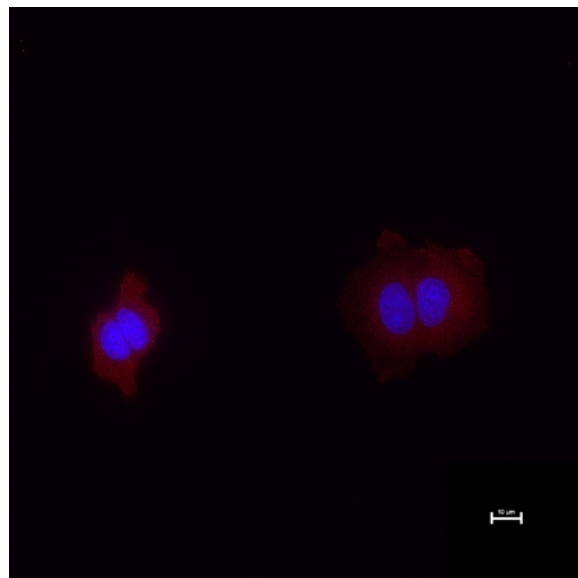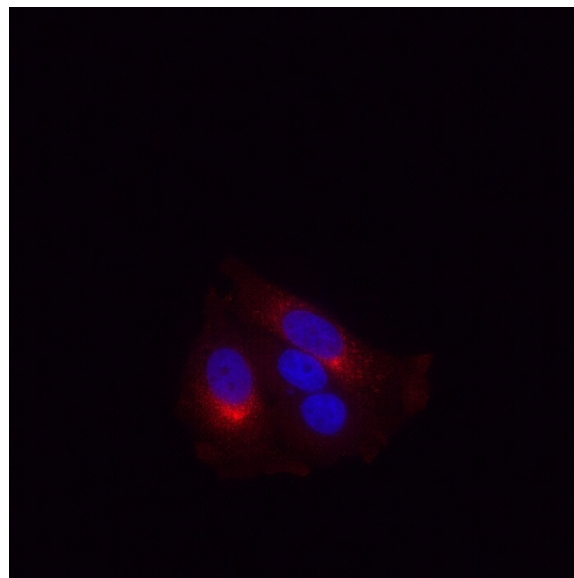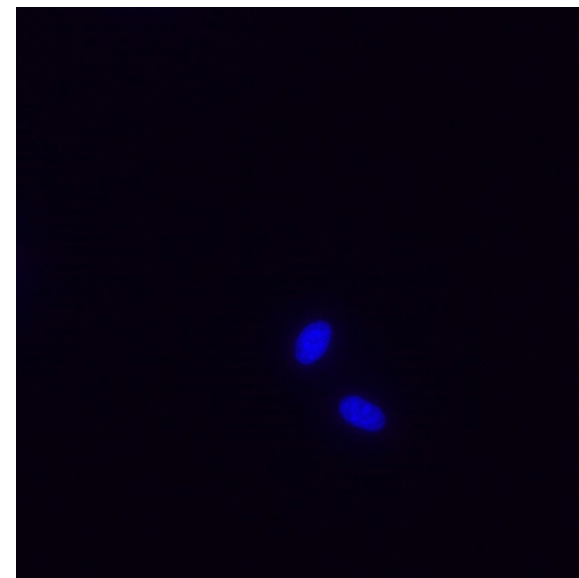

ApoE

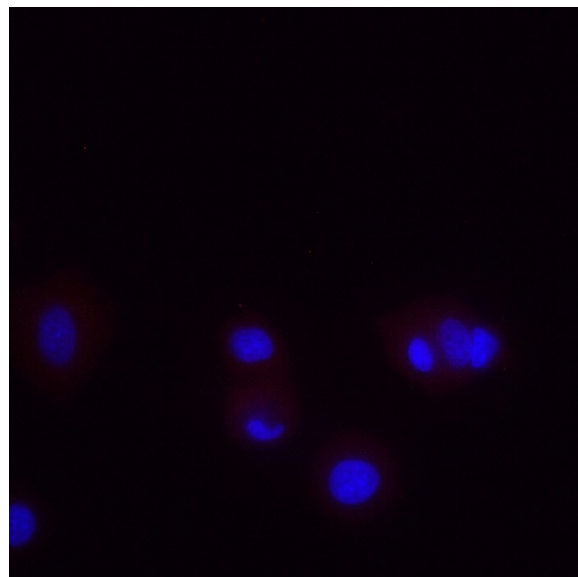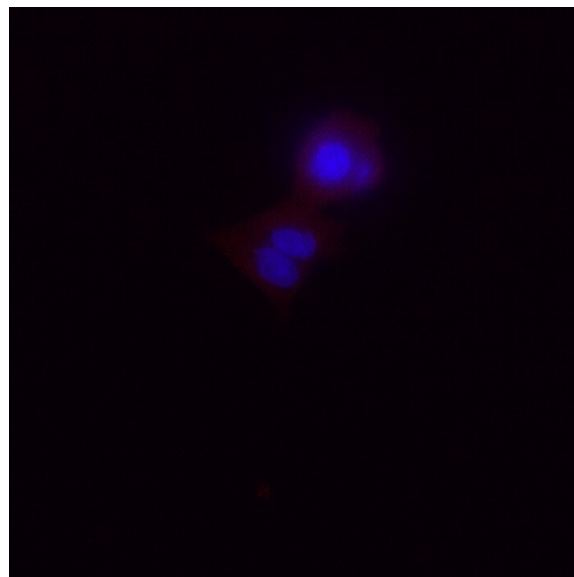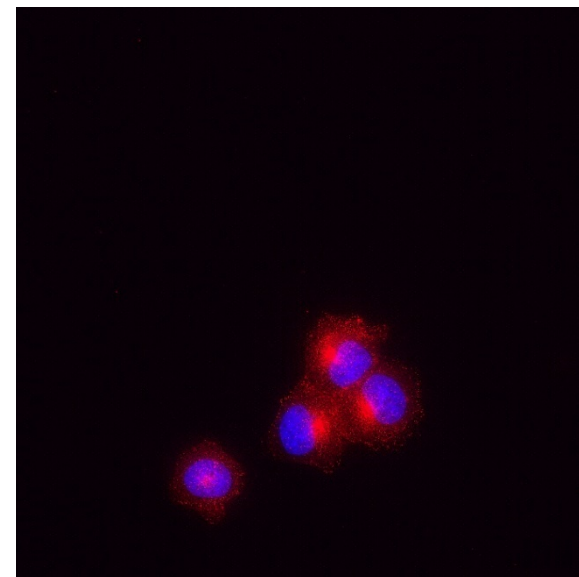

Supplement: Supplementary file 1 — Additional file 1:Supplementary Figure 1. Expression of ApoA-I and ApoE in Transfected MCF-7 and MDA-MB-231 Cells. A. mRNA levels in transfected MCF-7 and MDA-MB-231 cells. mRNA levels of APOA1 and APOE were quantified by RT-qPCR. ns: difference with control not significant. Significant difference compared to control cells: *P <0.05. B and C. Immunofluorescence detection of apoA-I and apoE in transfected MCF-7 and MDA-MB-231 cells. ApoA-I and apoE were detected by immunofluorescence using anti-apoA-I and anti-apoE antibodies and visualized by epifluorescence microscopy (scale = 10 μm). [file 13058_2020_1276_MOESM1_ESM.zip › Suppl Figure 1/Suppl Figure 1B.pdf]

MDA-MB-231

**Suppl. Figure 1C**

GFP

ApoA-I

ApoE

ApoA-I

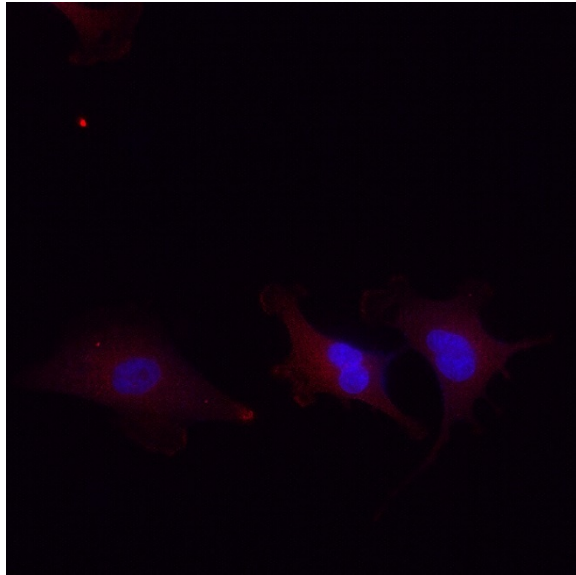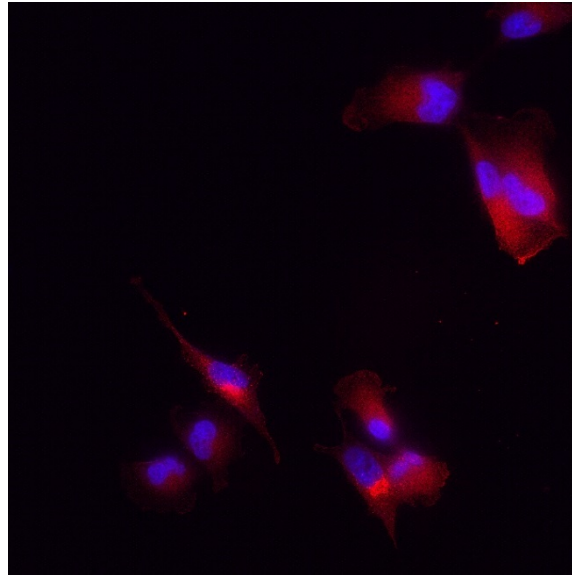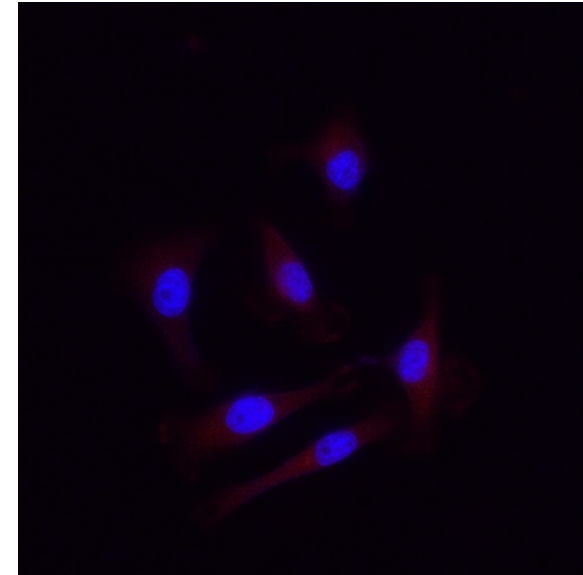

ApoE

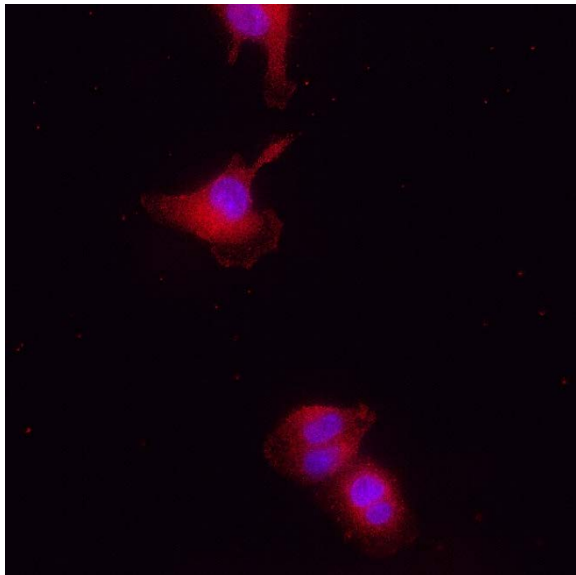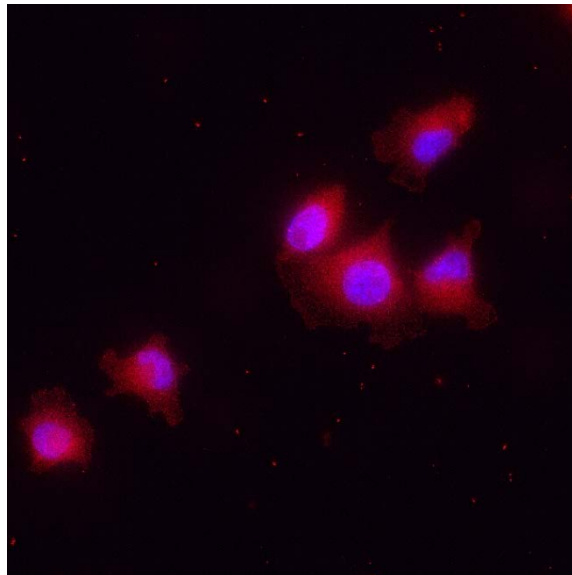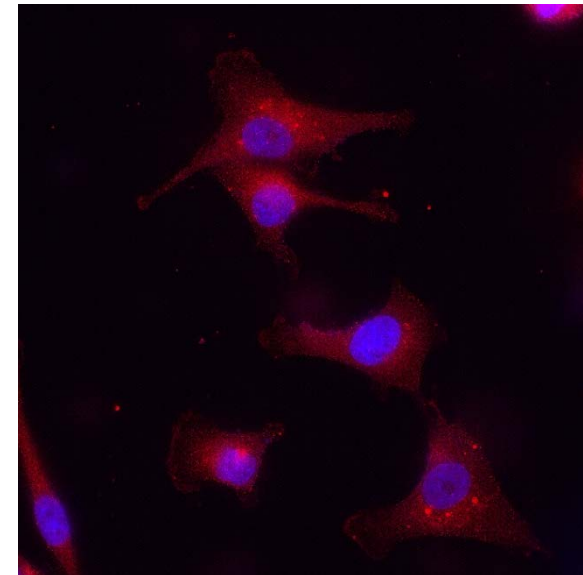

Supplement: Supplementary file 1 — Additional file 1:Supplementary Figure 1. Expression of ApoA-I and ApoE in Transfected MCF-7 and MDA-MB-231 Cells. A. mRNA levels in transfected MCF-7 and MDA-MB-231 cells. mRNA levels of APOA1 and APOE were quantified by RT-qPCR. ns: difference with control not significant. Significant difference compared to control cells: *P <0.05. B and C. Immunofluorescence detection of apoA-I and apoE in transfected MCF-7 and MDA-MB-231 cells. ApoA-I and apoE were detected by immunofluorescence using anti-apoA-I and anti-apoE antibodies and visualized by epifluorescence microscopy (scale = 10 μm). [file 13058_2020_1276_MOESM1_ESM.zip › Suppl Figure 1/Suppl Figure 1C.pdf]

## Suppl. Figure 2

### A. MCF-7

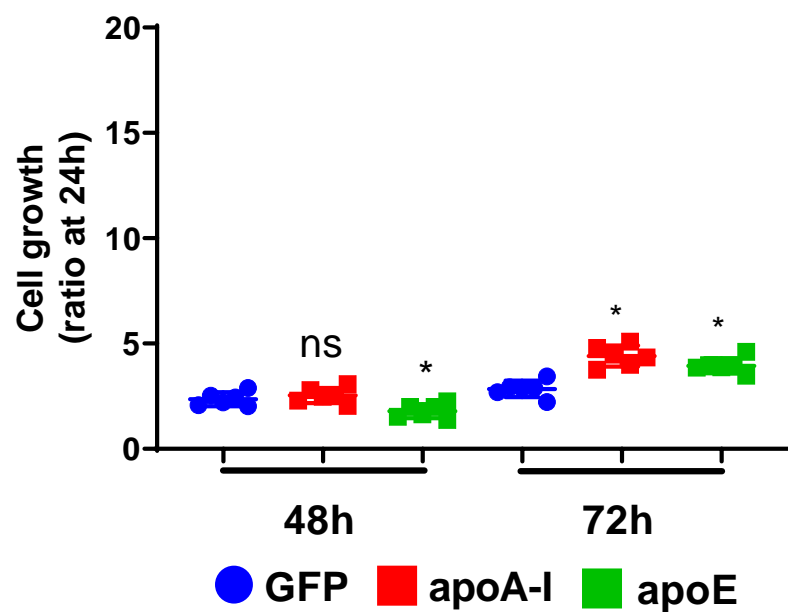

### B. MDA-MB-231

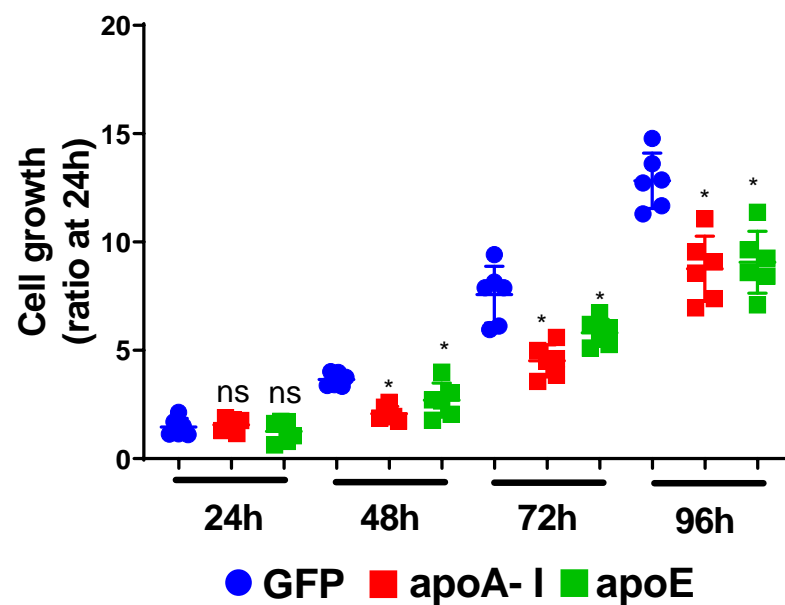

Supplement: Supplementary file 2 — Additional file 2:Supplementary Figure 2. ApoA-I and apoE increase proliferation of MCF-7 (A) but not of MDA-MB-231 (B) cells as determined by cell counting measurements. ns: difference with control not significant. Significant difference compared to control cells: *P <0.05. [file 13058_2020_1276_MOESM2_ESM.pdf]

# Suppl. Figure 3

Caveolin-1 Staining

MDA-MB-231

GFP

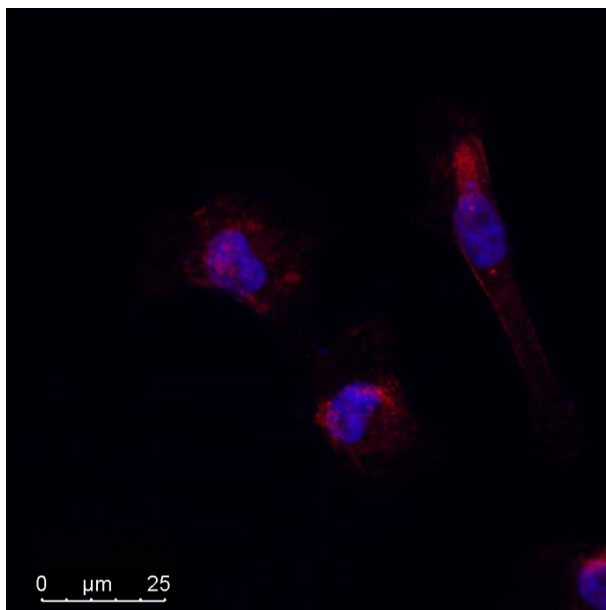

ApoA-I

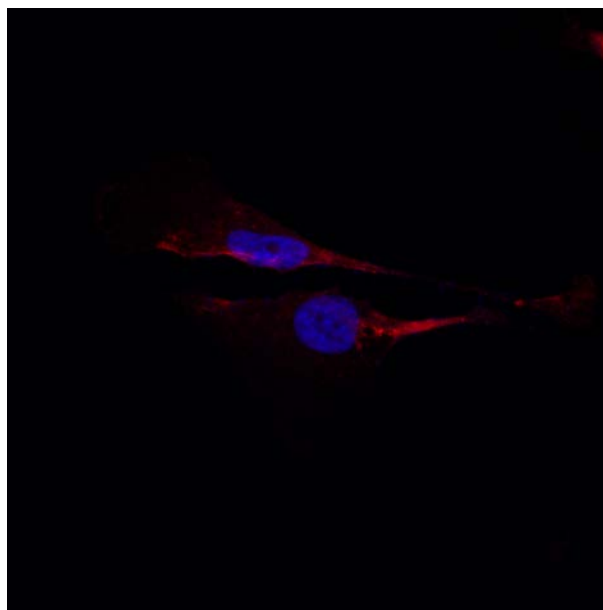

ApoE

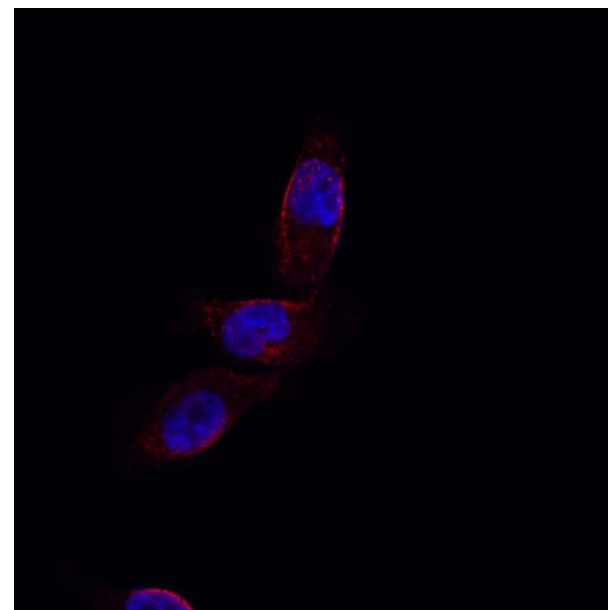

Supplement: Supplementary file 3 — Additional file 3:Supplementary Figure 3.: Immunostaining of caveolin-1 in MDA-MB-231 cells as evaluated by confocal microscopy. [file 13058_2020_1276_MOESM3_ESM.pdf]
